# Is the economic uncertainty– human health relationship nonlinear? An empirical analysis for the China

**DOI:** 10.1371/journal.pone.0293126

**Published:** 2023-12-07

**Authors:** Ziyu Guo, Muhammad Hafeez, Wenxin Wang, Md. Abdul Kaium, Ahmer Bilal, Israt Zahan

**Affiliations:** 1 Center for Disease Control and Prevention, Bao’an District, Shenzhen City, Guangdong Province, China; 2 Adnan Kassar School of Business, Lebanese American University, Beirut, Lebanon; 3 Institute of Business Management Sciences, University of Agriculture, Faisalabad, Pakistan; 4 School of Public Health, Shantou University, Shantou, Guangdong, People’s Republic of China; 5 Institute of Local Government Development, Shantou University, Shan‐Tou, People’s Republic of China; 6 Department of Marketing, University of Barishal, Barishal, Bangladesh; 7 School of Economics, Zhongnan University of Economics and Law, Wuhan, China; 8 Department of Public Administration, University of Barishal, Barishal, Bangladesh; Palestine Technical University - Kadoorie, STATE OF PALESTINE

## Abstract

The health costs of economic uncertainty always remain a major concern among policymakers of China. The theoretical and empirical literature on the economic uncertainty-human health nexus is still in its infancy stage. This study is firmly rooted in the economic uncertainty theory advanced by Baker, Bloom, & Davis. In this study, the primary objective of the analysis is to estimate the asymmetric impact of economic uncertainty on human health in China’s economy. In order to evaluate the short and long-run estimates of economic uncertainty on human health across various quantiles, we have employed the linear and nonlinear QARDL models. The linear QARDL model shows that the long-run relationship between economic uncertainty and the infant mortality rate is positive and significant at all quantiles, while the long-run relationship between economic uncertainty and the death rate is positive and significant at higher quantiles. The nonlinear QARDL model reveals that, in the long run, the relationship between the positive shock of economic uncertainty and the infant mortality rate is positive and significant at quantiles 0.30 to 0.95, while the long-run relationship between the positive shock of economic uncertainty and the death rate is positive and significant at higher quantiles. The relationship between the negative shock of economic uncertainty and the infant mortality rate is negative and significant at the highest quantiles, while the relationship between the negative shock of economic uncertainty and death rate is negative and significant at higher quantiles in the long run. The findings indicate a positive relationship between economic uncertainty in China and higher rates of infant mortality and death. Thus, adopting suitable policies for controlling economic uncertainty can help in improving human health in China.

## Introduction

Since the incident of the global financial crisis throughout the world, policymakers and scholars have become more interested in exploring the impact of economic uncertainty on human health [1,2]. But, empirical evidence regarding the impact of economic uncertainty on human health is still very scarce. Few studies have tried to explore the impact of economic uncertainty on human health by incorporating the role of unemployment, but these studies do not provide conclusive evidence [3]. The economic uncertainty concept is formless. Economic uncertainty is defined as uncertainty in consumers’, managers’, and policymakers’ minds regarding probable futures. Economic uncertainty also includes uncertainties regarding macro-phenomenon, e.g., GDP growth and micro-phenomenon e.g., firm growth rate, climate change, and war [4,5].

Macro and micro uncertainties both increase during recessions. Exogenous shocks such as oil price hikes, wars, and financial crises increase economic uncertainty [6]. Economic uncertainty appears to be endogenously increased during recessions because lower economic growth encourages greater micro and macro uncertainty [7,8]. Economic uncertainty leads to a reduction in the willingness of the firm to employ new labor, invest, and spend more on production [9]. Very few firms found themselves much more eager to innovate during periods of economic uncertainty. Economic uncertainty directly influences the investment and consumption pattern of the economy, which consequently generates a considerable effect on the whole business cycle [10]. From the consumption viewpoint, uncertainty stimulates risk-reluctant behavior that results in a decline in the current consumption of households [11]. It is anticipated that current consumption will be sacrificed for future savings. Caballero [12] described that consumption is negatively associated with economic uncertainty. Economic uncertainty triggers a significant reduction in investment patterns as well [13].

Empirical studies reveal that developed economies have to face the most severe incidence of uncertainty as compared with developing economies. China is also not free from economic uncertainty shocks [14]. China has the largest population in the world and economic uncertainty incidences are most likely to influence the population’s health at a large scale [15]. Due to globalization and the fastest-growing incorporation of China with the world economy, any incidence of uncertainty in any other economy also influences China. Dzielinski [16] proposed that financial relations and trade among nations play a fundamental part in explaining economic uncertainty.

Economic uncertainty influences every segment of the economy, but we are more interested in exploring the impact of economic uncertainty on human health [17]. No doubt, effective medical and health system planning would be beneficial for people’s physical health and disease risk declaration. The existing stock of studies has tried to explore the nexus between economic conditions and health-related behavior. Few studies reported a positive association between economic expansion and human health [18,19], while few studies reported that health deteriorates during an economic downturn [20]. Health effects are mixed and vary during different phases of the business cycle of the economy [21]. However, a lack of evidence is reported regarding the impact of economic uncertainty on human health. However, the decline in income may appear to increase the level of stress and anxiety among people which deteriorates health outcomes. Similarly, physical activity, drinking alcohol, and smoking activities may rise during the phase of recession and uncertainty [22]. A bulk of studies report the negative impact of the economic downturn on the mental health of people. Mental effects resultantly lead to economic stress that reports a negative impact on employment activities [23]. Extensive literature associates stress and anxiety with economic uncertainty and unemployment [24]. During the global financial crisis, the high rate of unemployment resulted in increased stress levels among people to a large extent [25,26]. Coope et al. [27] highlighted that economic uncertainty in Wales and England led to an increase in death rates in males, but a similar effect is not found for females. Various studies reported a similar effect in the case of EU countries that are facing severe financial challenges. Branas et al. [28] showed that the death rate increases during economic uncertainty in Greece.

Though the existing studies have explored the determinants of health, none of the studies has considered the impact of economic uncertainty on health. While there is a growing body of literature on the relationship between economic factors and health outcomes, there is limited research on the specific construct of economic uncertainty. The available studies on human health indicators have overlooked the impact of economic uncertainty on health outcomes. Very few studies are available exploring the nexus between economic policy uncertainty and human health in other regions of the world, but none of the studies is found in the case of China. Moreover, available studies have not tested the relationship in the nonlinear QARDL framework. The present study fills these gaps by employing the nonlinear QARDL approach to estimate the nonlinear effect of economic uncertainty on human health in China. The Chinese economy is the world’s most populated economy and needs proper health-oriented economic policies for the well-being of people. Moreover, it is a well-known fact that economic uncertainty disrupts the consumption pattern of households including health outcomes.

This study is significant for the following reasons. Firstly, while there is a considerable amount of research on the relationship between economic factors and health outcomes, there is a relative lack of research on the impact of economic uncertainty. This study helps to fill this gap by examining the nonlinear effect of economic uncertainty on human health in China. Secondly, the study’s findings can inform the development of public health policies that are better tailored to the needs of the population. Thirdly, the study can inform economic policies by highlighting the potential impact of economic uncertainty on health outcomes. Fourthly, China is the world’s most populous country, and its experiences can provide important insights into the relationship between economic uncertainty and health outcomes. The study’s findings can contribute to the global understanding of this relationship and inform policies in other countries. Lastly, by highlighting the impact of economic uncertainty on human health, the study underscores the importance of considering health outcomes in economic policy decisions. This can help policymakers develop more comprehensive and effective policies that take into account the broader impact on society.

The objective of this research study is to investigate the nonlinear impact of economic uncertainty on human health in China. The study makes various novel contributions to literature. Existing studies are exploring the asymmetric impact of economic factors on health outcomes but have not yet explored the role of economic uncertainty in determining health outcomes in China. Another important contribution is that our study is capturing human health through two proxy measures, e.g., death rate and infant mortality rate. In the baseline model infant mortality rate is used as a dependent variable, whereas, the death rate is used to check the robustness of the baseline model. Another deficiency of existing studies is that these studies do not take into account the non-normal distributed variables. However, our study takes into account the non-normal distribution of variables. Our study makes this novel contribution through the application of the nonlinear QARDL regression techniques. This new method makes this study quite unique and different from the existing one, as it reports the short-run and long-run impact of economic uncertainty on human health at lower, medium, and higher quantile ranges. Moreover, the nonlinear QARDL technique also captures the impact of positive and negative shocks in economic uncertainty on human health. The study provides useful guidelines to economic policymakers on how policy certainty can be improved to enhance human health.

## Model and methodology

Economic uncertainty triggers several health-related issues such as anxiety and stress [29]. The COVID-19 pandemic is a recent example of the negative impact of economic uncertainty on health. Following the COVID-19 pandemic and the financial crisis of 2008, psychologists and economists have focused on investigating the effect of economic uncertainty on human health and well-being [30]. Various studies reported that recession triggers mental illness and suicide instances in society [31]. The studies added theoretical reasoning as the economic uncertainties report a detrimental impact on families’ income and health structure. A prominent example is Mishel’s [32] theory of uncertainty and health, which postulates that uncertainty stems from a lack of information, unpredictability, vagueness, and ambiguity.

To explore the long-run and short-run asymmetries among economic uncertainty and human health variables, we have employed the QARDL technique proposed by Cho et al [33]. The QARDL method is an econometric approach used to analyze time series data, particularly when examining the relationships between variables with nonlinearity and asymmetric effects. It is an extension of the standard ARDL model, which is often used for modeling the long-run and short-run relationships between variables. The key feature of the QARDL method is its ability to capture how the relationship between variables may vary across different quantiles of the data distribution. In other words, it allows for the analysis of how the effects of variables change at different points along the distribution of the dependent variable. The QARDL approach is dominant over linear models based on various reasons. The first edge of adopting this technique is that it takes into account the locational asymmetries in which factors and findings may be conditional on the dependent variable. Due to this reason, QARDL is considered more appropriate, as the linear ARDL technique cannot capture the asymmetric association among variables. Another edge is that the QARDL approach considers the long-run dynamics as well as short-run dynamics over different quantile ranges [34]. The QARDL method provides more efficient and reliable estimates of the coefficients compared to other quantile regression methods [35]. One of the primary benefits of the QARDL method is its ability to handle non-normal distributions, which are common in economic data. It allows for analyzing relationships across different quantiles of the data distribution, providing insights into how variables may impact the outcome differently at various points along the distribution. Moreover, in this approach, the Wald test is used to detect the time-varying reliability of variables across quantiles. In the study carried out by Vandoros et al. [36], a model signifying the link between economic uncertainty on human health is presented as follows:

$${\text{Health}}_{\text{t}}=\mu +\overset{\text{n}1}{\underset{\text{i}=1}{\sum }}\;\sigma_{{\text{Health}}_{\text{i}}}{\text{Health}}_{\text{t}-\text{i}}+\overset{\text{n}2}{\underset{\text{i}=0}{\sum }}\;\sigma_{{\text{EU}}_{\text{i}}}{\text{EU}}_{\text{t}-\text{i}}+\overset{\text{n}3}{\underset{\text{i}=0}{\sum }}\;\sigma_{{\text{GDP}}_{\text{i}}}{\text{GDP}}_{\text{t}-\text{i}}+\overset{\text{n}4}{\underset{\text{i}=0}{\sum }}\;\sigma_{{\text{FD}}_{\text{i}}}{\text{FD}}_{\text{t}-\text{i}}+\overset{\text{n}5}{\underset{\text{i}=0}{\sum }}\;\sigma_{{\text{HE}}_{\text{i}}}{\text{HE}}_{\text{t}-\text{i}}+\varepsilon_{\text{t}}$$ (1)

Where ε_t_ is explained as Health_t_ -E[Healtht/Ft − 1] with Ft– 1 is the smallest σ–field made by (EU_t_, GDP_t_, FD_t_, HE_t_, EU_t-1_, GDP_t-1_, FD_t-1_, HE_t-1_}, and n1….n5 represents the lag orders for model variables indicated by Schwarz information criterion (SIC). Eq (1) infers that economic uncertainty, GDP per capita, financial development, and health expenditure are represented by EU_t_, GDP_t_, FD_t_, HE_t_, respectively, while Health_t_ represents health outcomes. Following Cho et al. [33] approach, we have to reformat basic Eq (1) in the quantile ARDL format:

$$\begin{array}{l} {\text{Q}}_{{\text{Health}}_{\text{t}}}=\mu \left(\tau \right)+\;\overset{\text{n}1}{\underset{\text{i}=1}{\sum }}\;\sigma_{{\text{Health}}_{\text{i}}}\left(\tau \right){\text{Health}}_{\text{t}-\text{i}}+\;\overset{\text{n}2}{\underset{\text{i}=0}{\sum }}\;\sigma_{{\text{EU}}_{\text{i}}}\left(\tau \right){\text{EU}}_{\text{t}-\text{i}}+\;\overset{\text{n}3}{\underset{\text{i}=0}{\sum }}\;\sigma_{{\text{GDP}}_{\text{i}}}\left(\tau \right){\text{GDP}}_{\text{t}-\text{i}}\\ +\overset{\text{n}4}{\underset{\text{i}=0}{\sum }}\;\sigma_{{\text{FD}}_{\text{i}}}\left(\tau \right){\text{FD}}_{\text{t}-\text{i}}+\overset{\text{n}5}{\underset{\text{i}=0}{\sum }}\;\sigma_{{\text{HE}}_{\text{i}}}\left(\tau \right){\text{HE}}_{\text{t}-\text{i}}+\varepsilon_{\text{t}}\left(\tau \right) \end{array}$$ (2)

The equation ε_t_(τ) = Health_t_ − QHealth_t_(τ/Ft −1) defines εt(τ) as the difference between Health_t_ and QHealth_t_(τ/Ft −1), where 0 < τ < 1 represents the quantile level. To account for potential serial correlation in Eq (2), we can employ a nonlinear QARDL model. Our analysis primarily focuses on the assumption of nonlinearity, as mentioned earlier. To facilitate this, we utilized the partial sum procedure to divide the variable of economic uncertainty (EU) into positive and negative components. The EU variables were decomposed for nonlinear analysis.


$${\text{EU}}^{+}{}_{\text{t}}=\;\overset{\text{t}}{\underset{\text{n}=1}{\sum }}\;{\Delta \text{EU}}^{+}{}_{\text{t}}=\overset{\text{t}}{\underset{\text{n}=1}{\sum }}\;\text{max}({\Delta \text{EU}}^{+}{}_{\text{t}},0)$$ (3a)

$${\text{EU}}^{-}{}_{\text{t}}=\overset{\text{t}}{\underset{\text{n}=1}{\sum }}\;{\Delta \text{EU}}^{-}{}_{\text{t}}=\overset{\text{t}}{\underset{\text{n}=1}{\sum }}\;\text{min}({\Delta \text{EU}}^{\;-}{}_{\text{t}},\;0)$$ (3b)

Next, we revisit Eq (2) and substitute the positive and negative variations of EU to obtain:

$$\begin{array}{l} {\text{Q}}_{{\Delta \text{Health}}_{\text{t}}}=\mu +{\rho \text{Health}}_{\text{t}-1}+\pi^{+}{}_{\text{EU}}{\text{EU}}^{+}{}_{\text{t}-1}+\pi^{-}{}_{\text{EU}}{\text{EU}}^{-}{}_{\text{t}-1}+\pi_{\text{GDP}}{\text{GDP}}_{\text{t}-\text{k}}+\pi_{\text{FD}}{\text{FD}}_{\text{t}-\text{k}}+\pi_{\text{HE}}{\text{HE}}_{\text{t}-\text{k}}\\ +\;\overset{\text{n}1}{\underset{\text{i}=1}{\sum }}\;\delta_{{\text{Health}}_{\text{i}}}{\Delta \text{Health}}_{\text{t}-\text{i}}+\;\overset{\text{n}2}{\underset{\text{k}=0}{\sum }}\;\delta^{+}{}_{{\text{EU}}_{\text{i}}}{\Delta \text{EU}}^{+}{}_{\text{t}-\text{i}}+\;\overset{\text{n}3}{\underset{\text{k}=0}{\sum }}\;\delta^{-\text{on}\;\text{GE}\;\text{een}\;\text{ods}.\text{Contrariwise},}{}_{{\text{EU}}_{\text{i}}}{\Delta \text{EU}}^{-}{}_{\text{t}-\text{i}}\\ +\overset{\text{n}4}{\underset{\text{i}=0}{\sum \;}}\delta_{{\text{GDP}}_{\text{i}}}{\Delta \text{GDP}}_{\text{t}-\text{i}}+\overset{\text{n}5}{\underset{\text{i}=0}{\sum }}\;\delta_{{\text{FD}}_{\text{i}}}{\Delta \text{FD}}_{\text{t}-\text{i}}+\overset{\text{n}6}{\underset{\text{i}=0}{\sum }}\;\delta_{{\text{HE}}_{\text{i}}}{\Delta \text{HE}}_{\text{t}-\text{i}}+\varepsilon_{\text{t}}\left(\tau \right) \end{array}$$ (4)

The QARDL-ECM structure can be extended to incorporate the nonlinear QARDL framework as outlined in Eq (2). This issue can be avoided by projecting ε_t_ onto specific factors. Therefore, the error correction approach for representing the QARDL framework is described below:

$$\begin{array}{l} {\text{Q}}_{{\Delta \text{Health}}_{\text{t}}}=\mu \left(\tau \right)+\rho \left(\tau \right)({\text{Health}}_{\text{t}-1}-\pi^{+}{}_{\text{EU}}\left(\tau \right){\text{EU}}^{+}{}_{\text{t}-1}-\pi^{-}{}_{\text{EU}}\left(\tau \right){\text{EU}}^{-}{}_{\text{t}-1}-\pi_{\text{GDP}}\left(\tau \right){\text{GDP}}_{\text{t}-1}\\ -\pi_{\text{FD}}\left(\tau \right){\text{FD}}_{\text{t}-1}-\pi_{\text{HE}}\left(\tau \right){\text{HE}}_{\text{t}-1})+\;\overset{\text{n}1}{\underset{\text{i}=1}{\sum }}\;\delta_{{\text{Health}}_{\text{i}}}\left(\tau \right){\Delta \text{Health}}_{\text{t}-\text{i}}+\;\overset{\text{n}2}{\underset{\text{i}=0}{\sum }}\;\delta^{+\;\text{on}\;\text{GE}\;\text{een}\;\text{ods}.\text{Contrariwise},}{}_{{\text{EU}}_{\text{i}}}\left(\tau \right){\Delta \text{EU}}^{+}{}_{\text{t}-\text{i}}\\ +\;\overset{\text{n}3}{\underset{\text{i}=0}{\sum }}\;\delta^{-\;\text{on}\;\text{GE}\;\text{een}\;\text{ods}.\text{Contrariwise},}{}_{{\text{EU}}_{\text{i}}}\left(\tau \right){\Delta \text{EU}}^{-}{}_{\text{t}-\text{i}}+\overset{\text{n}4}{\underset{\text{i}=0}{\sum }}\;\delta_{{\text{GDP}}_{\text{i}}}\left(\tau \right){\Delta \text{GDP}}_{\text{t}-\text{i}}+\;\overset{\text{n}5}{\underset{\text{i}=0}{\sum }}\;\delta_{{\text{FD}}_{\text{i}}}\left(\tau \right){\Delta \text{FD}}_{\text{t}-\text{i}}\\ +\overset{\text{n}6}{\underset{\text{i}=0}{\sum }}\;\delta_{{\text{HE}}_{\text{i}}}\left(\tau \right){\Delta \text{HE}}_{\text{t}-\text{i}}+\varepsilon_{\text{t}}\left(\tau \right) \end{array}$$ (5)

To measure the combined short-term impact of lagged health (Health) on current outcomes, we calculate $\delta^{*}\sum_{\text{j}=1}^{\text{n}}\;\delta_{\text{j}}$. The cumulative short-run dynamics of EU^+^_t_, EU^−^_t_, GDP_t_, FD_t_, and HE_t_ are denoted by $\delta^{*}\sum_{\text{j}=0}^{\text{n}2}\;\delta_{\text{j}},\delta^{*}\sum_{\text{j}=0}^{\text{n}3}\;\delta_{\text{j}},\delta^{*}\sum_{\text{j}=0}^{\text{n}4}\;\delta_{\text{j}},\pi^{*}\sum_{\text{j}=0}^{\text{n}5}\;\pi_{\text{j}},\delta^{*}\sum_{\text{j}=0}^{\text{n}6}\;\delta_{\text{j}}$. Similarly, the underlying long-run relationships among economic uncertainty, GDP, financial development, and health expenditures are described using the coefficients $\pi^{+}{}_{\text{EU}}*=-\frac{\pi^{+}\text{EU}}{\text{p}},\;\pi^{-}{}_{\text{EU}}*=-\frac{\pi^{-}\text{EU}}{\text{p}},\;\pi_{\text{GDP}}*=-\frac{\pi \text{GDP}}{\text{p}},\;\pi_{\text{FD}}*=-\frac{\pi \text{FD}}{\text{p}}$, and $\pi_{\text{HE}}*=-\frac{\pi \text{HE}}{\text{p}}$. Eq (5) estimates that the parameter (ρ) associated with the Health parameter should be significantly negative. The Wald test has been employed to examine the short- and long-term nonlinear effects of the EU on Health. If the null hypotheses π^+^_EU_ = π^−^_EU_ (δ^+^_EU_ = δ^−^_EU_) are rejected by the Wald test, long-term (short-term) asymmetries will be identified.

## Data and descriptive analysis

To investigate the effect of economic uncertainty on human health in China from 1990 to 2020, this study employed six variables. Two of them are dependent variables, such as infant mortality rate and death rate. The infant mortality rate is the number of deaths of infants under one year of age per 1,000 live births in a given year. The death rate is the number of deaths per 1,000 people in a given year. The independent variable includes economic uncertainty. This is a measure of the level of uncertainty or volatility in the economy and can be measured using a variety of indicators, such as stock market volatility, inflation rates, and exchange rate fluctuations. Following Ullah et al. [37], our study measures it through inflation variation from its mean values. Economic uncertainty can have a significant impact on a range of economic and social outcomes, including health outcomes. GDP, financial development, and health expenditures are used as control variables. GDP per capita is a commonly used measure of a country’s economic output per person and is often used as an indicator of a country’s standard of living [38]. GDP per capita in our study is taken constant at 2015 US$. Financial development is a measure of the level of development of a country’s financial system and can be measured using a range of indicators, such as the number of financial institutions, the level of financial intermediation, and the level of financial inclusion. Following Yang et al. [39], financial development in this study is measured by domestic credit to the private sector as % of GDP. Health expenditure is the total amount of money spent on healthcare in a given year and can be measured at the national, regional, or individual level. Health expenditure in our study is measured by current health expenditures as % of GDP.

The data sources of all variables are World Bank, except economic uncertainty. The variable’s details are reported in the appendix (Table A) in S1 Appendix. Results for descriptive statistics are given in Table 1. The mean of IMR, DR, EU, GDP, FD, and HE are 2.896, 6.783, 0.016, 8.136, 4.756, and 4.256 years, respectively. In order to confirm the normality of the data series, the study used J-B test. The results of J-B test confirm that the null hypothesis of linearity is rejected for all variables, which allows us to use the QARDL approach for regression purposes.

**Table 1:** Results of descriptive statistics.

	Mean	Median	Max	Min	S.D	Skewness	Kurtosis	Jarque-Bera	Prob.
IMR	2.896	2.965	3.755	1.681	0.675	-0.264	1.679	10.45	0.005
DR	6.783	6.702	7.143	6.398	0.285	0.003	1.256	15.71	0.000
EU	0.016	-1.682	20.90	-5.565	5.457	2.298	8.179	247.6	0.000
GDP	8.136	8.128	9.247	6.790	0.758	-0.114	1.728	8.631	0.013
FD	4.756	4.717	5.258	4.428	0.213	0.257	2.212	4.574	0.102
HE	4.256	4.245	5.370	3.416	0.547	0.318	1.997	7.291	0.026

## Empirical results and discussion

The pre-requisite condition for applying the QARDL model is to investigate the integration order of the variables through unit root tests. The purpose of these unit root tests is to make the variables stationary. Therefore, DF-GLS, Zivot, and Andrew (ZA) structural break unit root tests were applied to find out the stationary variables. The results are described in Table 2. It can be seen from the results that just EU is stationary at a level while all other variables, i.e., IMR, DR, GDP, FD, and HE, are stationary at the 1^st^ difference in both unit root tests.

**Table 2:** Results of unit-root tests.

	DF-GLS		ZA				Decision	
	I(0)	I(1)	I(0)	Break date	I(1)	Break date	DF-GLS	ZA
IMR	-1.023	-4.032***	-2.354	2013 Q2	-5.365***	2019 Q1	**I(1)**	**I(1)**
DR	-1.256	-1.789*	-7.654***	2005 Q1			**I(1)**	**I(0)**
EU	-2.721***		-6.587***	1994 Q1			**I(0)**	**I(0)**
GDP	-1.487	-1.987**	-2.366	2010 Q2	-6.256***	1993 Q1	**I(1)**	**I(1)**
FD	1.366	-2.754***	-2.023	2008 Q1	-6.352***	1993 Q1	**I(1)**	**I(1)**
HE	-0.578	-2.654***	-2.012	2008 Q1	-5.365***	1993 Q3	**I(1)**	**I(1)**

In Table 3, the results of QARDL show that the constant term coefficient is significant, while the ECM values are negative and significant at most quintile. The coefficient is significantly negative and describes convergence toward long-run equilibrium. However, long-run coefficient results reveal that EU is positively associated with the IMR and it has a negative effect on human health. Furthermore, the finding indicates that there is a high magnitude of EU at the higher quantiles. On the other hand, GDP, FD, and HE are negatively linked to IMR, with a higher magnitude at higher quantiles. This implies that GDP, FD, and HE have a positive impact on human health. Our result aligns with the findings of Dhrifi [40] and Rana et al. [41]. Our finding infers that higher GDP allows for increased investment in healthcare infrastructure, such as hospitals, clinics, and healthcare facilities. Higher GDP leads to improved maternal healthcare services. With increased economic resources, countries can invest in prenatal care, skilled birth attendants, and emergency obstetric care. Access to quality maternal healthcare reduces complications during pregnancy and childbirth, which can have a direct impact on infant survival rates. The finding of Cole [42] is in line with this result. Higher GDP is often accompanied by improvements in living standards, income levels, and social infrastructure. These socioeconomic factors contribute to overall well-being and can indirectly affect infant mortality rates.

**Table 3:** Infant mortality rate estimates (QARDL).

	ECM	Constant	Long-run estimates				Short-run estimates						
	ρ(τ)	μ(τ)	π_EU_(τ)	π_GDP_(τ)	π_FD_(τ)	π_HE_(τ)	δ0_EU_(τ)	δ1_EU_(τ)	δ0_GDP_(τ)	δ1_GDP_(τ)	δ0_FD_(τ)	δ1_FD_(τ)	δ_HE_(τ)
0.05	-0.242***	11.15***	0.008***	-0.581***	-0.725***	-0.059	0.001	0.000	-0.331***	-0.324***	-0.067***	-0.060***	-0.001
	(-6.983)	(22.71)	(4.053)	(-22.12)	(-4.705)	(-1.084)	(1.570)	(1.151)	(-2.595)	(-2.511)	(-9.700)	(-5.987)	(-0.545)
0.10	-0.254***	11.33***	0.007***	-0.584***	-0.746***	-0.062	0.001	0.000	-0.314**	-0.309*	-0.062***	-0.058***	-0.001
	(-5.911)	(19.30)	(2.887)	(-19.17)	(-3.995)	(-0.980)	(1.257)	(0.955)	(-2.021)	(-1.957)	(-7.369)	(-4.556)	(-0.465)
0.20	-0.278***	11.66***	0.004*	-0.554***	-0.853***	-0.077	0.001	0.001	-0.315*	-0.311*	-0.052***	-0.050***	-0.000
	(-6.468)	(16.66)	(1.872)	(-13.90)	(-3.806)	(-1.082)	(1.039)	(0.825)	(-1.627)	(-1.701)	(-4.946)	(-3.311)	(-0.107)
0.30	-0.274***	12.75***	0.008**	-0.749***	-0.804***	-0.013	0.001	0.001	-0.396***	-0.397***	-0.043***	-0.049***	-0.001
	(-6.764)	(39.13)	(2.326)	(-11.74)	(-6.216)	(-0.337)	(1.286)	(1.639)	(-2.696)	(-2.632)	(-3.545)	(-3.044)	(-0.351)
0.40	-0.261***	12.93***	0.011***	-0.796***	-0.815***	-0.078	0.002	0.001	-0.413***	-0.415***	-0.076*	-0.085**	-0.000
	(-5.925)	(37.78)	(4.755)	(-27.42)	(-6.081)	(-1.476)	(1.011)	(1.457)	(-3.286)	(-3.214)	(-1.923)	(-2.011)	(-0.266)
0.50	-0.225***	12.11***	0.010***	-0.839***	-0.503***	-0.007	0.001	0.001	-0.314*	-0.312*	-0.072*	-0.073*	-0.001
	(-4.494)	(24.20)	(6.072)	(-28.82)	(-3.089)	(-0.175)	(1.372)	(0.925)	(-1.905)	(-1.829)	(-1.759)	(-1.951)	(-0.665)
0.60	-0.231***	12.45***	0.011***	-0.836***	-0.593***	-0.026	0.001	0.000	-0.229*	-0.225*	-0.048*	-0.044*	-0.002
	(-4.294)	(24.20)	(7.278)	(-21.43)	(-3.614)	(-0.802)	(1.162)	(0.637)	(-1.674)	(-1.862)	(-1.844)	(-1.807)	(-1.246)
0.70	-0.245***	12.37***	0.010***	-0.844***	-0.557***	-0.022	0.001	0.000	-0.212*	-0.208*	-0.043*	-0.038*	-0.002
	(-4.223)	(21.88)	(8.038)	(-20.92)	(-3.115)	(-0.652)	(1.094)	(0.471)	(-1.699)	(-1.692)	(-1.750)	(-1.943)	(-1.232)
0.80	-0.237***	11.47***	0.007***	-0.866***	-0.297*	-0.002	0.001	0.000	-0.217**	-0.213*	-0.039*	-0.036*	-0.001
	(-4.401)	(19.58)	(4.500)	(-18.08)	(-1.686)	(-0.059)	(1.250)	(0.584)	(-2.001)	(-1.900)	(-1.922)	(-1.818)	(-0.680)
0.90	-0.252***	11.09***	0.006***	-0.867***	-0.230*	-0.007	0.001	0.000	-0.198**	-0.195**	-0.041*	-0.038*	-0.002
	(-5.076)	(19.16)	(3.875)	(-14.83)	(-1.782)	(-0.194)	(1.397)	(0.599)	(-2.097)	(-2.004)	(-1.990)	(-1.739)	(-1.611)
0.95	-0.242***	11.08***	0.006***	-0.865***	-0.233*	-0.009	0.000	0.000	-0.112**	-0.105*	-0.020**	-0.015**	-0.003
	(-3.669)	(24.17)	(4.954)	(-12.28)	(-1.727)	(-0.290)	(0.551)	(0.453)	(-1.982)	(-1.892)	(-2.002)	(-1.984)	(-0.898)

**Note**: The t-stat is in parentheses.

***p<0.01;

**p<0.05;

*p<0.1.

This outcome supports the empirical report by Shobande [43], who noted that financial development enhances healthcare access for families. Access to financial services enables individuals to better manage healthcare expenses, such as medical treatments, prenatal care, and childbirth services. Improved financial resources and affordability of healthcare can lead to better access to quality healthcare, ultimately reducing infant mortality rates. This finding also infers that financial development facilitates investment in healthcare infrastructure, including hospitals, clinics, and medical technologies. Access to financial resources allows for the expansion and improvement of healthcare facilities, which can positively impact maternal and child health outcomes. Financial development is often associated with socioeconomic development, including increased income levels, reduced poverty rates, and improved living standards. These factors indirectly influence infant mortality rates by improving overall socioeconomic conditions. Our findings align with the results reported by Akinlo & Sulola [44] who noted that health expenditure is a significant factor, and its impact on infant mortality is also influenced by various other socioeconomic, cultural, and healthcare system factors. The short-run finding shows that the recent trend in human health in China is insignificantly and positively influenced by the EU. This means that the EU did not influence IMR in short-run. Similarly, HE has also no effect on IMR in short-run. While GDP and FD have a negative and significant impact on IMR.

The QARDL result of the death rate model is reported in Table 4. In the baseline model infant mortality rate is used as a dependent variable, whereas, the death rate is used to check the robustness of the baseline model. The findings show that the constant term is significant and positive at all quantiles. Furthermore, the ECM coefficient is also significantly negative at most quantiles, which indicates convergence toward the long-run equilibrium. The long-run coefficient results indicate that EU has positively and significantly associated with the death rate at only higher quantiles, such as 0.60–0.95. It means that as the EU increases the death rate at higher quantiles. Our findings are backed by various studies. For instance, Vandoros et al. [36] denoted that economic uncertainty reports a negative impact on human health in the form of anxiety and stress. Godinić & Obrenovic [3] also report a negative association between economic uncertainty and human health. The study denotes that economic uncertainty reports a negative psychological impact. Economic uncertainty creates anxiety, discomfort, provokes vulnerability, fear, and lack of decision-making among people. Iheoma [45] reports a negative association between economic uncertainty and health expenditures, which indicates the vulnerability of people in response to an economic shock. Another study done by Corro Ramos et al. [46] reported that economic uncertainty shrinks the size of public health expenditures that negatively influence the health outcomes of people. Kim [47] also denoted that economic uncertainty reduces government revenues which leads to a significant reduction in the allocated budget of the health sector. Due to a decline in health expenditures, overall health outcomes decline. Matusitz & Musambira [48] highlighted that if economic uncertainty is prolonged, it will deteriorate the economic structure of the economy including the health sector. The finding also infers that economic uncertainty leads to unemployment that reduces the per capita income of people, hence, making them less able to obtain good quality health services.

**Table 4:** Death rate estimates (QARDL).

	ECM	Constant	Long-run estimates				Short-run estimates			
	ρ(τ)	μ(τ)	π_EU_(τ)	π_GDP_(τ)	π_FD_(τ)	π_HE_(τ)	δ_EU_(τ)	δ_GDP_(τ)	δ_FD_(τ)	δ_HE_(τ)
0.05	-0.121	3.179***	0.020	-0.881***	-0.663**	-0.152	0.003	-0.010	-0.002	-0.004
	(-0.033)	(3.561)	(1.185)	(-9.314)	(-2.428)	(-1.139)	(1.509)	(-0.663)	(-0.141)	(-0.229)
0.10	-0.201	4.851***	0.009	-0.735***	-0.834***	-0.052	0.001	-0.010	-0.005	-0.009
	(-1.116)	(6.994)	(1.154)	(-16.51)	(-3.464)	(-0.822)	(1.571)	(-0.722)	(-0.370)	(-0.495)
0.20	-0.194	5.292***	0.007	-0.707***	-0.898**	-0.025	0.001	-0.012	-0.009	-0.010
	(-0.465)	(4.419)	(1.511)	(-11.55)	(-2.108)	(-0.239)	(1.143)	(-1.031)	(-0.753)	(-0.692)
0.30	-0.332	4.645***	0.007	-0.592***	-0.487**	-0.100**	0.000	-0.032	-0.003	-0.031
	(-0.889)	(9.551)	(1.075)	(-11.98)	(-2.446)	(-2.298)	(0.791)	(-1.070)	(-0.095)	(-0.869)
0.40	-0.277***	4.900***	0.006	-0.603***	-0.534**	-0.123**	0.000	-0.051***	-0.030**	-0.051***
	(-3.088)	(8.388)	(1.071)	(-11.44)	(-2.385)	(-2.084)	(0.701)	(-5.450)	(-2.062)	(-4.876)
0.50	-0.279***	4.962***	0.005	-0.639***	-0.562**	-0.169**	0.000	-0.049***	-0.033**	-0.051***
	(-3.381)	(7.866)	(1.530)	(-10.39)	(-2.339)	(-2.372)	(0.552)	(-5.237)	(-2.373)	(-4.978)
0.60	-0.280***	4.518***	0.003**	-0.649***	-0.408*	-0.250***	0.000	-0.048***	-0.037***	-0.054***
	(-3.651)	(4.528)	(2.021)	(-8.344)	(-1.752)	(-2.668)	(0.313)	(-4.981)	(-2.831)	(-5.194)
0.70	-0.276***	5.984***	0.003**	-0.461***	-0.448*	-0.163**	0.000	-0.046**	-0.043**	-0.058***
	(-2.943)	(5.082)	(2.316)	(-4.764)	(-1.781)	(-2.048)	(1.300)	(-2.515)	(-2.411)	(-2.840)
0.80	-0.276***	6.023***	0.006*	-0.407***	-0.360**	-0.160**	0.000	-0.046**	-0.043**	-0.058***
	(-2.943)	(5.493)	(1.679)	(-5.869)	(-2.010)	(-2.133)	(1.300)	(-2.515)	(-2.411)	(-2.840)
0.90	-0.272***	6.598***	0.007*	-0.392***	-0.493*	-0.114**	0.001*	-0.061***	-0.167***	-0.102***
	(-3.188)	(7.802)	(1.918)	(-7.021)	(-1.795)	(-2.026)	(1.857)	(-5.485)	(-3.091)	(-5.007)
0.95	-0.266***	6.660***	0.008*	-0.387***	-0.495**	-0.115**	0.002*	-0.060***	-0.159***	-0.096***
	(-2.948)	(9.797)	(1.836)	(-8.824)	(-2.334)	(-2.260)	(1.713)	(-5.926)	(-3.709)	(-6.041)

**Note**: The t-stat is in parentheses.

***p<0.01;

**p<0.05;

*p<0.1.

Our finding is reinforced by Park & Mosley [49] who offer supporting evidence. The study denotes that economic uncertainty shrinks government revenue, which indirectly consumption, production, and investment to slow down under uncertainty that increases unemployment. Due to an upsurge in unemployment, health expenses shrink drastically. Moreover, a reduction in government revenue tends to reduce government health expenditures that result in the deterioration of human health in developing economies. Hornung & Bandelow [50] also reported that economic uncertainty tends to increase unemployment which leads to a reduction in per capita health expenditure. Studies highlighted that people in developing economies are expected to be influenced by fear of losing a job, insecurity, reduction in income, social isolation, and unavailability of basic healthcare services. Claveria [51] reported that economic uncertainty triggers suicide counts.

Regarding control variables, GDP, FD, and HE are negatively linked with death rates while showing higher magnitudes in higher quantiles. It denotes that GDP, FD, and HE have a positive impact on human health by reducing the death rate. While our outcomes are consistent with the findings of Liang & Tussing [52] and Bhupatiraju [53]. This infers that GDP has a negative impact on the death rate by improving healthcare infrastructure and socioeconomic factors. The findings indicate that financial development increases access to banking services and financial inclusion can enhance healthcare access for individuals and communities. Financial development is often associated with socioeconomic development, including increased income levels, reduced poverty rates, and improved living standards. These socioeconomic factors have a positive impact on health outcomes and can indirectly influence the death rate. Financial development contributes to economic growth, job creation, and income generation, leading to improved access to nutritious food, better living conditions, and reduced exposure to health risks. These factors can contribute to overall improvements in health and a decrease in the death rate. Our results are in line with the findings reported by Coccia [54]. Increased health expenditure leads to the expansion and improvement of healthcare infrastructure. This expansion enhances the availability and accessibility of healthcare services for the population. This finding also infers that health expenditure contributes to medical research, innovation, and the development of new treatments and technologies. The short-run analysis depicts that EU has no impact on death rates except the last two quantiles, i.e., 0.90 and 0.95. This infers that EU has significantly increased the death rate at only the highest quantiles. Similarly, GDP, FD, HE negatively and significantly impact on death rate at medium and higher quantiles, such as 0.40–0.95.

The results presented in Table 5 demonstrate the findings of the nonlinear QARDL analysis. The coefficient of the constant term is found to be significant across all quantiles, indicating its importance. Additionally, the ECM values exhibit a negative and significant relationship with all quantiles. These negative ECM coefficients indicate a convergence toward long-run equilibrium. Furthermore, the long-run coefficient results reveal that a positive shock in the EU is significantly and positively associated with the IMR within a wide range of quantiles (0.30 to 0.95). This suggests that a positive shock in the EU has a detrimental effect on human health. Conversely, a negative shock in the EU is found to have a significant negative impact on IMR only at the highest quantiles (0.80 to 0.95). This implies that a negative shock in the EU tends to improve human health in the long run, specifically at higher quantiles. In contrast, GDP is negatively correlated with IMR at quantiles 0.70 to 0.95, indicating a positive impact on human health in the long run. Similarly, FD exhibits a negative relationship with IMR across all quantiles, suggesting a positive influence on human health in the long run. On the other hand, HE does not exhibit a significant impact on IMR in the long run. Regarding the short-run analysis, a positive shock in the EU is found to result in a positive increase in IMR within the quantiles 0.80 to 0.95, confirming a deterioration in human health. Conversely, a negative shock in EU has no influence on IMR in the short run. Similarly, HE also has no effect on IMR in the short run. However, GDP and FD have a negative and significant impact on IMR only at higher quantiles, suggesting a positive effect on human health in the short run.

**Table 5:** Infant mortality rate estimates (Nonlinear QARDL).

(τ)	ECM	Constant	Long-run estimates					Short-run estimates					
	ρ(τ)	μ(τ)	π^+^_EU_(τ)	π^−^_EU_(τ)	π_GDP_(τ)	π_FD_(τ)	π_HE_(τ)	δ^+^_EU_(τ)	δ^−^_EU_(τ)	δ0_GDP_(τ)	δ1_GDP_(τ)	δ_FD_(τ)	δ_HE_(τ)
0.05	-0.378***	9.925***	0.005	0.033	-0.274	-0.171***	-0.059	0.211	0.094	-0.142	-0.009	-0.035	-0.013
	(-2.969)	(2.635)	(1.045)	(0.463)	(-0.045)	(-1.689)	(-0.466)	(0.021)	(1.034)	(-1.112)	(-0.431)	(-0.515)	(-0.168)
0.10	-0.248**	7.247**	0.006	0.035	-0.326	-0.232**	-0.076	0.321	0.099	-0.205	-0.012	-0.068	-0.016
	(-2.416)	(1.978)	(1.244)	(0.524)	(-0.231)	(-2.012)	(-0.501)	(0.451)	(1.110)	(-1.232)	(-0.593)	(-0.644)	(-0.275)
0.20	-0.215***	4.192**	0.006	0.044	-0.385	-0.387**	-0.089	0.456	0.103	-0.225	-0.014	-0.074	-0.018
	(-4.261)	(2.499)	(1.387)	(0.668)	(-0.658)	(-2.123)	(-0.620)	(0.612)	(1.233)	(-1.325)	(-0.640)	(-0.711)	(-0.353)
0.30	-0.226***	8.805***	0.007*	0.052	-0.512	-0.456**	-0.099	0.645	0.113	-0.253	-0.018	-0.079	-0.023
	(-4.117)	(4.454)	(1.698)	(0.705)	(-0.785)	(-2.355)	(-0.759)	(0.754)	(1.387)	(-1.456)	(-0.757)	(-0.874)	(-0.466)
0.40	-0.246***	9.591***	0.008**	0.067	-0.611	-0.564***	-0.108	0.712	0.131	-0.274	-0.021	-0.081	-0.027
	(-3.444)	(3.847)	(2.021)	(0.866)	(-0.897)	(-2.658)	(-0.869)	(0.803)	(1.088)	(-1.489)	(-0.824)	(-0.901)	(-0.540)
0.50	-0.301***	10.21***	0.008**	0.077	-0.712	-0.687***	-0.114	0.526	0.140	-0.301	-0.023	-0.095	-0.032
	(-5.544)	(4.256)	(2.325)	(0.918)	(-1.023)	(-2.897)	(-0.994)	(1.026)	(0.914)	(-1.521)	(-1.120)	(-1.029)	(-0.683)
0.60	-0.275***	12.93***	0.010**	0.080	-0.756	-0.789***	-0.120	0.427	0.167	-0.343	-0.027	-0.078	-0.037
	(-4.342)	(3.845)	(2.557)	(1.045)	(-1.614)	(-3.254)	(-1.036)	(1.257)	(0.877)	(-1.578)	(-1.297)	(-1.253)	(-0.591)
0.70	-0.266***	13.45***	0.009**	0.056	-0.845*	-0.987***	-0.141	0.332	0.183	-0.373*	-0.032	-0.069	-0.042
	(-4.217)	(3.387)	(2.335)	(1.236)	(-1.898)	(-2.587)	(-1.154)	(1.595)	(0.745)	(-1.678)	(-1.104)	(-1.451)	(-0.413)
0.80	-0.236***	14.66***	0.007**	0.045*	-0.897**	-1.325**	-0.162	0.296*	0.147	-0.405*	-0.041	-0.056*	-0.034
	(-3.876)	(5.992)	(2.214)	(1.679)	(-2.023)	(-2.365)	(-1.210)	(1.867)	(0.652)	(-1.758)	(-1.027)	(-1.684)	(-0.378)
0.90	-0.234***	15.14***	0.007**	0.034*	-0.957**	-0.795**	-0.184	0.129**	0.126	-0.351**	-0.052	-0.049*	-0.023
	(-3.806)	(3.745)	(2.111)	(1.849)	(-2.325)	(-2.144)	(-1.394)	(2.086)	(0.515)	(-2.012)	(-0.852)	(-1.724)	(-0.358)
0.95	-0.392***	16.87***	0.006*	0.027**	-0.897**	-0.678**	-0.208	0.048**	0.102	-0.279**	-0.062	-0.033**	-0.021
	(-3.516)	(5.256)	(1.897)	(2.120)	(-2.456)	(-2.015)	(-1.296)	(2.261)	(0.428)	(-2.321)	(-0.578)	(-2.011)	(-0.254)

**Note**: The t-stat is in parentheses.

***p<0.01;

**p<0.05;

*p<0.1.

The findings of the nonlinear QARDL analysis for the death rate model are presented in Table 6. The constant term coefficient is found to be significant across all quantiles, emphasizing its importance. Additionally, the ECM values display a negative and significant relationship with high quantiles (0.70 to 0.95), indicating a convergence toward long-run equilibrium. Furthermore, the long-run coefficient results reveal a significant and positive association between a positive shock in the EU and the DR at high quantiles (0.60 to 0.95). This suggests that a positive shock in the EU has a detrimental effect on human health. Conversely, a negative shock in the EU exhibits a significant negative impact on DR only at the highest quantiles (0.70 to 0.95), indicating an improvement in human health in the long run, particularly at higher quantiles. In contrast, GDP and FD are negatively correlated with DR across all quantiles, indicating a positive impact on human health in the long run. Similarly, HE shows a negative relationship with DR at quantiles 0.50 to 0.95, suggesting a positive influence on human health in the long run. Regarding the short-run analysis, a positive shock in the EU leads to a positive increase in DR within the quantiles 0.70 to 0.95, confirming a deterioration in human health. Conversely, a negative shock in the EU does not influence DR in the short run. GDP and FD have a negative and significant impact on DR only at higher quantiles, indicating a positive effect on human health in the short run. However, HE does not have any effect on DR in the short run. The results of the Wald test are presented in Table 7, confirming the presence of asymmetry in economic uncertainty across higher quantiles in the long run in both models. However, in the short run, the Wald test reported only nonlinear relationships in EU in only IMR model.

**Table 6:** Death rate estimates (Nonlinear QARDL).

(τ)	ECM	Constant	Long-run estimates					Short-run estimates				
	ρ(τ)	μ(τ)	π^+^_EU_(τ)	π^−^_EU_(τ)	π_GDP_(τ)	π_FD_(τ)	π_HE_(τ)	δ^+^_EU_(τ)	δ^−^_EU_(τ)	δ_GDP_(τ)	δ_FD_(τ)	δ_HE_(τ)
0.05	-0.213	3.996***	0.021	0.008	-0.911**	-0.434*	-0.005	0.008	0.121	-0.006	0.008	-0.002
	(-0.189)	(4.022)	(0.313)	(0.209)	(-2.495)	(-1.951)	(-0.804)	(0.604)	(0.144)	(-0.189)	(0.071)	(-0.110)
0.10	-0.245	3.368***	0.024	0.009	-0.821**	-0.550**	-0.008	0.011	0.101	-0.008	0.006	-0.003
	(-0.201)	(4.235)	(0.457)	(0.333)	(-2.232)	(-2.122)	(-1.201)	(0.811)	(0.247)	(-0.214)	(0.096)	(-0.124)
0.20	-0.278	3.155***	0.029	0.011	-0.789**	-0.644**	-0.012	0.013	0.098	-0.010	0.003	-0.003
	(-0.341)	(4.566)	(0.651)	(0.456)	(-2.114)	(-2.327)	(-1.354)	(0.975)	(0.310)	(-0.327)	(0.101)	(-0.245)
0.30	-0.321	2.417***	0.034	0.013	-0.745**	-0.711**	-0.023	0.016	0.071	-0.012	0.001	-0.004
	(-0.389)	(4.894)	(0.742)	(0.521)	(-2.005)	(-2.455)	(-1.537)	(1.214)	(0.432)	(-0.444)	(0.245)	(-0.354)
0.40	-0.345	2.231***	0.042	0.015	-0.711*	-0.789***	-0.029	0.022	0.067	-0.014	-0.002	-0.005
	(-0.412)	(5.321)	(0.897)	(0.854)	(-1.897)	(-2.653)	(-1.634)	(1.456)	(0.513)	(-0.564)	(-0.365)	(-0.456)
0.50	-0.378	2.051***	0.049	0.016	-0.657**	-0.845***	-0.033*	0.028	0.057	-0.016	-0.005	-0.006
	(-0.842)	(5.654)	(1.298)	(1.295)	(-2.231)	(-2.805)	(-1.852)	(1.523)	(0.651)	(-0.833)	(-0.546)	(-0.521)
0.60	-0.412	3.066***	0.054*	0.019	-0.615**	-0.687***	-0.045**	0.032	0.048	-0.017	-0.009	-0.006
	(-1.312)	(6.325)	(1.723)	(1.475)	(-2.365)	(-2.987)	(-2.001)	(1.625)	(0.730)	(-1.325)	(-0.875)	(-0.625)
0.70	-0.378*	3.319***	0.062**	0.021*	-0.524**	-0.521**	-0.054**	0.021*	0.059	-0.014	-0.012	-0.007
	(-1.712)	(5.322)	(2.042)	(1.782)	(-2.558)	(-2.456)	(-2.326)	(1.789)	(0.678)	(-1.554)	(-1.352)	(-0.725)
0.80	-0.324**	3.766***	0.042**	0.023*	-0.445***	-0.489**	-0.062**	0.018*	0.069	-0.013*	-0.015*	-0.006
	(-1.949)	(4.325)	(2.327)	(1.879)	(-2.987)	(-2.235)	(-2.476)	(1.897)	(0.578)	(-1.784)	(-1.875)	(-0.812)
0.90	-0.298**	4.011***	0.034**	0.020**	-0.546***	-0.423**	-0.045**	0.014*	0.081	-0.011*	-0.018**	-0.005
	(-2.013)	(3.689)	(2.546)	(1.989)	(-3.211)	(-2.121)	(-2.540)	(2.013)	(0.475)	(-1.929)	(-2.154)	(-1.245)
0.95	-0.278**	4.231***	0.022***	0.017**	-0.621***	-0.387**	-0.032***	0.011**	0.089	-0.009**	-0.023**	-0.004
	(-2.456)	(3.355)	(2.687)	(2.125)	(-3.254)	(-1.989)	(-2.678)	(2.325)	(0.354)	(-2.112)	(-2.568)	(-1.345)

**Note**: The t-stat is in parentheses.

***p<0.01;

**p<0.05;

*p<0.1.

**Table 7:** Results of Wald test.

	Infant mortality rate		Death rate	
	Long-run	Short-run	Long-run	Short-run
0.05	0.154	1.354	1.023	1.254
0.10	0.199	1.414	1.352	1.325
0.20	0.234	1.541	1.425	1.498
0.30	0.248	1.982	1.564	2.035
0.40	1.220	2.222	1.857	3.201
0.50	2.101	2.023	2.032	2.345
0.60	3.874*	3.652*	2.321	2.012
0.70	4.652**	4.552*	3.987*	1.985
0.80	5.698***	4.879**	4.321*	1.678
0.90	6.854***	5.652****	4.689**	1.568
0.95	8.698***	6.655***	6.025***	1.254

## Conclusion and policy recommendations

One of the most complicated but basic concepts in our life is development. Despite some new indicators of well-being, Gross Domestic Product per person is still known to be among the most used indicators to represent well-being. Health infrastructure in a country is largely dependent overall wealth of a nation, which GDP represents. Therefore, we can say that affluence and health go side by side. However, economic uncertainty can significantly impact the country’s per capita income, which may hurt human health because people no longer afford good health services. Researchers have tried to examine the link between GDP and human health in the past, but very few might have observed the nonlinear impact of economic uncertainty on human health. Therefore, the primary objective of the analysis is to estimate the impact of nonlinear economic uncertainty on human health in China.

In order to evaluate the short and long-run estimates of economic uncertainty on human health across various quantiles, we have employed the linear and nonlinear QARDL models. The linear QARDL model reveals that the long-run relationship between economic uncertainty and the infant mortality rate is positive and significant at all quantiles, while the long-run relationship between economic uncertainty and the death rate is positive and significant at higher quantiles. These findings suggest that the higher the economic uncertainty in China higher the infant mortality and death rates will be. The nonlinear QARDL reveals that the positive shock in the EU is significantly and positively associated with the IMR within a wide range of quantiles (0.30 to 0.95) in the long run. Conversely, a negative shock in the EU is found to have a significant negative impact on IMR only at the highest quantiles (0.80 to 0.95). The long-run coefficient results reveal a significant and positive association between a positive shock in the EU and the DR) at high quantiles (0.60 to 0.95). Conversely, a negative shock in the EU exhibits a significant negative impact on DR only at the highest quantiles (0.70 to 0.95), indicating an improvement in human health in the long run, particularly at higher quantiles. Conversely, the long-run impact of GDP, financial development, and health expenditures on infant mortality and death rates appear to be negative. In the short run, the results are insignificant and inconclusive. The long-run asymmetric impact of economic uncertainty, GDP, financial development, and health expenditures are confirmed in the infant mortality rate model, while in the case of the death rate model, the asymmetric impacts are confirmed for GDP and health expenditures.

These findings may help policymakers and other interested parties make decisions about future policy direction. In times of economic unpredictability, individuals are less likely to act in the interest of society because they are preoccupied with meeting their immediate material needs and those of their families. Huge long-term impacts on health and finances may result from crises for the whole society and not only for individuals. Therefore, social protection programs and public health care efforts for people may benefit from a deeper understanding of the psychological mechanics of crises and risks. By comprehending these factors, more effective techniques may be used to reduce the severity of the economic effects and stop the spread of mental and physical disorders. Moreover, through financial development, more funds can be made available for investment in health infrastructure by the private sector. Similarly, policymakers can increase public sector investment in health-related infrastructure by increasing health spending at the government level.

The government should strengthen social safety net programs, such as unemployment insurance, social assistance, and health insurance, to mitigate the negative impact of economic uncertainty on vulnerable populations. The government should work to promote financial stability and reduce economic uncertainty through policies that encourage sustainable economic growth and stable financial markets. This could include regulations to prevent financial crises, prudent fiscal and monetary policies, and efforts to promote social and economic stability. The government should invest in healthcare infrastructure and services to improve the accessibility and quality of healthcare, particularly in rural areas. This could include building more healthcare facilities, increasing the number of healthcare professionals, and improving the availability of medical supplies and equipment. The government should invest in public health education programs to promote healthy behaviors and prevent disease. This could include providing information on healthy eating, exercise, and disease prevention strategies. The government should promote mental health awareness and reduce the stigma around mental health issues, particularly in times of economic uncertainty. This could include providing education and resources for mental health support and encouraging the development of community-based mental health services.

To effectively work towards attaining the sustainable development goals (SDGs), the following suggestions can be considered. Ensure equitable access to quality healthcare services, particularly for marginalized communities. Enhance healthcare infrastructure, increase healthcare professionals’ availability, and expand health insurance coverage to reduce the barriers to accessing healthcare during economic downturns. Allocate resources to strengthen public health infrastructure, including disease surveillance systems, emergency preparedness, and response mechanisms. This will enable proactive measures to be taken in addressing health challenges arising from economic uncertainties, such as epidemics or other health crises. Economic uncertainty can lead to increased stress, anxiety, and mental health issues. Develop and implement comprehensive mental health support programs, including awareness campaigns, counseling services, and community-based support systems to address mental health needs in times of economic instability. Encourage collaboration between government entities, healthcare organizations, non-profit organizations, and private sectors to jointly address the impact of economic uncertainty on human health. Pool resources, knowledge, and expertise to implement sustainable interventions that can effectively mitigate health risks during economic challenges. By implementing these suggestions, stakeholders can take practical steps to address the impact of economic uncertainty on human health in China and work towards achieving the SDGs related to health and well-being.

Despite useful policy suggestions, this study contains some limitations. Firstly, the study is done at the aggregate level due to the non-availability of data at the provincial level. Future studies should consider the provinces of China in the analysis. Secondly, our study measures economic uncertainty through inflation variations. Future studies should use some other proxy variables such as trade uncertainty and economic policy uncertainty. This study can be extended to other economies of the world. Moreover, future studies should include other aspects in analysis like digital technologies, education, and income level that can affect health outcomes. Finally, future studies should also examine the economic uncertainty-human health nexus by using time series models i.e. SVAR model, TVP-VAR model, and Hybrid TVP-VAR models.

## Supporting information

S1 Dataset (XLSX)

S1 Appendix (DOCX)
