# Supplementary material for: Is the economic uncertainty– human health relationship nonlinear? An empirical analysis for the China
Source: PLoS One. 2023 Dec 7;18(12):e0293126. doi: 10.1371/journal.pone.0293126 (PMC10703211; doi:10.1371/journal.pone.0293126)
Supplement: S1 Appendix — (DOCX) [file pone.0293126.s002.docx]

**Appendix**

**Table A: Variables description and sources**

| **Variables** | **Symbol** | **Definitions** | **Sources** |
| --- | --- | --- | --- |
| Infant mortality rate | IMR | Mortality rate, infant (per 1,000 live births) | WDI |
| Death rate | DR | Death rate, crude (per 1,000 people) | WDI |
| Economic uncertainty | EU | Inflation variation from its mean values | Author calculation |
| GDP per capita | GDP | GDP per capita (constant 2015 US$) | WDI |
| Financial development | FD | Domestic credit to private sector (% of GDP) | WDI |
| Health expenditure | HE | Current health expenditure (% of GDP) | WDI |
